# Patient Characteristics Associated with HCV Treatment Adherence, Treatment Completion, and Sustained Virologic Response in HIV Coinfected Patients

**DOI:** 10.1155/2011/903480

**Published:** 2011-10-26

**Authors:** Glenn Wagner, Karen Chan Osilla, Jeffrey Garnett, Bonnie Ghosh-Dastidar, Laveeza Bhatti, Matthew Bidwell Goetz, Mallory Witt

**Affiliations:** ^1^Health Unit, RAND Corporation, 1776 Main Street, P.O. Box 2138, Santa Monica, CA 90407, USA; ^2^Health Unit, RAND Corporation, Arlington, VA 22202, USA; ^3^AIDS Healthcare Foundation, Los Angeles, CA 90028, USA; ^4^Infectious Diseases Section, Greater Los Angeles Veterans Administration, Los Angeles, CA 90073, USA; ^5^Los Angeles Biomedical Research Institute at Harbor-UCLA Medical Center, Los Angeles, CA 90509, USA

## Abstract

*Background*. Hepatitis C (HCV) treatment efficacy among HIV patients is limited by poor treatment adherence and tolerance, but few studies have examined the psychosocial determinants of treatment adherence and outcomes. *Methods*. Chart abstracted and survey data were collected on 72 HIV patients who had received pegylated interferon and ribavirin to assess correlates of treatment adherence, completion, and sustained virologic response (SVR). *Results*. Nearly half (46%) the sample had active psychiatric problems and 13% had illicit drug use at treatment onset; 28% reported <100% treatment adherence, 38% did not complete treatment (mostly due to virologic nonresponse), and intent to treat SVR rate was 49%. Having a psychiatric diagnosis was associated with nonadherence, while better HCV adherence was associated with both treatment completion and SVR. *Conclusions*. Good mental health may be an indicator of HCV treatment adherence readiness, which is in turn associated with treatment completion and response, but further research is needed with new HCV treatments emerging.

## 1. Introduction

Over 30% of all HIV patients are estimated to be coinfected with hepatitis C virus (HCV), and liver disease is now a leading cause of death in this population [1–3]. Yet very few (<10%) coinfected patients actually receive HCV therapy [4–7], in part because of limited treatment efficacy and anticipated difficulty with treatment adherence. The advent of pegylated-interferon (PEG-IFN) therapy in combination with ribavirin (RBV) has led to dramatic improvements in the efficacy of HCV treatment, but response rates remain limited, particularly among coinfected patients; sustained virologic response (SVR), defined as an undetectable HCV viral load six months after the completion of treatment, is roughly 20–45% among coinfected patients [8–13], and is inversely associated with severity of immune suppression [14]. 

The success of treatment is impeded by high rates of premature treatment discontinuations and poor adherence [15]. Rates of premature HCV treatment discontinuation among coinfected patients is nearly double that of HCV monoinfected patients, ranging from 25% in highly controlled clinical trials [8] to 50% in community-based studies [12, 13], with most of this dropout attributed to treatment side effects or early virologic nonresponse. HCV treatment adherence research has largely focused on whether treatment is completed and on amount of dose reductions in PEG-IFN and RBV [16–20]. We are aware of only a few published studies that have reported on patient adherence to HCV therapy. Fumaz and colleagues found that over 98% of HIV coinfected patients self-reported taking all doses of PEG-IFN/RBV in the prior 2 weeks at weeks 12, 24, and 48 of treatment [21]. In a clinical trial sample of HCV monoinfected subjects, Smith and colleagues found that at least 95% of subjects self-reported taking all doses of PEG-IFN throughout the course of treatment, while the proportion of patients who reported recent missed doses of RBV increased from 10–25% over the course of treatment [22]. Similarly, in a cross-sectional study of both HCV monoinfected and HIV coinfected patients, Weiss et al. found that only 8% reported missing a dose of PEG-IFN in the past month, while 21% had missed a dose of RBV in the past week [23]. 

Specific medical (e.g., anemia, neutropenia) [24, 25] and psychiatric [26, 27] variables render patients vulnerable to greater toxicities associated with PEG-IFN/RBV, which may in turn predict treatment adherence and tolerance, but correlates of HCV treatment adherence have seldom been examined. In contrast, a large number of predictors of HIV antiretroviral therapy (ART) adherence have been identified, including variables related to patient demographics, psychosocial functioning, and disease characteristics [28–30]. Similar variables may be associated with HCV treatment adherence, but studies are needed to establish these relationships. 

In this report we document findings from a study of HIV coinfected patients receiving HCV care at one of three HIV clinics in Los Angeles. We assessed the frequency and correlates of adherence, treatment completion, and SVR among coinfected clients who had received PEG-IFN/RBV, either currently or in the past.

## 2. Methods

### 2.1. Sample

The participating HIV clinics were located at the Greater Los Angeles Veterans Administration (VA) Medical Center, Harbor-UCLA Medical Center, and AIDS Healthcare Foundation (AHF). During the 4-month study enrollment period, the study coordinator at each site performed a chart review of all patients attending the clinic for a routine visit to identify those who were coinfected with HCV and otherwise eligible. Patients were asked to provide consent for a brief survey and abstraction of data from their medical chart as they were waiting to be seen by their provider. Only patients who had actually received interferon-based HCV treatment were included in the analysis for this paper. The study protocol was approved by the Institutional Review Boards at RAND and the individual clinics.

### 2.2. Measures

We abstracted data that were closest and prior to the date HCV treatment was started. In addition, participants were asked to complete a brief questionnaire. 

*Demographic and background characteristics* that were assessed included age, gender, race/ethnicity, education, employment, and relationship status.

*HIV and HCV medical characteristics* that were abstracted included CD4 cell count, HIV viral load, and whether or not the patient was on HIV antiretroviral therapy at the start of treatment. With regard to HCV and liver disease, measures included HCV viral load and genotype. 

*Adherence to HCV and HIV medication regimens *were assessed with single visual analog scales. For each regimen type, respondents were asked to rate their overall adherence on a scale of 0% to 100% to show their “best guess about how much of your prescribed HCV (HIV) medication you took during the time you were (have been) on treatment.” Respondents were only asked to rate their adherence to ART if they were on ART at the time of the survey, whereas the HCV adherence question was asked if the respondent had ever been on HCV therapy (past or current). Due to the highly skewed response distributions, and evidence that self-reports overestimate adherence by 10–20% compared to objective measures [30], these variables were dichotomized using 100% in one category, and <100% as the other. 

*Clinic attendance* was assessed by asking respondents to report whether or not they had missed any scheduled clinic appointments over the past 6 months. A dichotomous variable was then created to represent none versus any missed appointments. 

*Psychosocial readiness *for treatment adherence focused on mental health and substance use. We extracted from chart data whether the patient had a diagnosis of depression or any other psychiatric disorder, and any documented problems with alcohol or illicit drug use (marijuana, cocaine, heroin, crystal methamphetamine or other) over the past 6 months, at the start of HCV treatment. We also extracted whether the patient was receiving any form of psychiatric treatment (e.g., psychotropic medication, counseling).

### 2.3. Data Analysis

Descriptive statistics were used to examine the frequency distributions of variables. We used bivariate statistics (independent 2-tailed *t*-tests, Chi Square tests) to examine correlates of HCV treatment adherence, treatment completion, and sustained virologic response in separate analyses. Multivariate analysis was planned but not performed due to so few variables being associated with the outcome variables in the bivariate analysis; this is likely a result of the small sample sizes and limited statistical power.

## 3. Results

We enrolled a sample of 173 patients in the study, of whom 127 (73%) had been offered HCV treatment at some point, and 72 (42%) had received PEG-IFN/RBV treatment (7 others were about to start treatment; 48 patients who had been recommended treatment, opted to refuse or defer it). Findings regarding factors associated with provider recommendation of treatment [31], and patient acceptance and initiation of treatment [32], have been reported elsewhere. In this paper we report analyses on the data from the subgroup of 72 treated patients. 

Among the 72 patients who had received HCV treatment, nearly all (94%) were male, mean age was 48.1 years (SD = 9.2), 51% were non-White (including 31% African American), 57% had received at least some college education, 33% were employed, and 23% were currently in a relationship. Regarding medical characteristics, the vast majority (91%) were on ART, mean CD4 count at time of study entry was 534 (SD = 234), and 79% had an undetectable HIV viral load. Most (71%) had an HCV genotype of 1 or 4, and 43% had an HCV viral load less than 800,000 copies. Nearly half (46%) had documentation in their clinic chart of any psychiatric problem at study entry, and 13% had documentation of any illicit drug use in the 6 months prior to study entry.

### 3.1. Dose Adherence

Among the 72 treated patients, the average length of time between treatment initiation and study enrollment was 3.2 years (range: 64 days to 10.5 years). All patients received once-a-week PEG-IFN injections and twice daily oral RBV. The prescribed medication dosage was not collected. Mean adherence to the PEG-IFN/RBV regimen over the entire period that the patient was on treatment (past or current) was 93.8% (SD = 15.3; range: 10–100%); 72% (*n* = 49) reported 100% (“good”) adherence or not missing any doses of either PEG-IFN or RBV, with the other 19 (28%) reporting less than 100% (“poor”) adherence; 4 participants did not respond to the adherence question, each of whom did not successfully complete the full course of treatment, which could suggest that they are more likely to have been poor adherers. In bivariate analysis, compared to good adherers, poor adherers were more likely to have any active psychiatric diagnosis at the time of treatment initiation (67% versus 38%; *P* < .05) (see Table 1); no other variables were significantly associated with HCV treatment adherence. 

### 3.2. Treatment Completion

Of the 72 treated patients, 43 (60%) completed a full course of prescribed treatment (33 completed 48 weeks, five completed 24–32 weeks and five completed 52 to 72 weeks), 3 were still on treatment at the time of the survey, and 26 (38% of the 69 with determined treatment status) prematurely discontinued treatment. Of the 26 who discontinued treatment, the primary reasons included virologic nonresponse (*n* = 14) and adverse events (*n* = 10); the other two patients discontinued due to unspecified reasons. Excluding the patients who were currently on treatment, we compared the 43 who successfully completed treatment with the 26 who discontinued treatment. The only variable that was significantly associated with treatment completion in bivariate analysis was HCV treatment adherence; 79% of those who completed the full course of treatment reported good HCV treatment adherence, compared to 55% of those who prematurely discontinued treatment (*P* < .05) (see Table 2).

### 3.3. Treatment Virologic Response

Among the 43 participants who completed HCV treatment, 32 achieved an SVR six months after treatment completion, while 7 had detectable HCV RNA; the SVR status of the other 4 patients was pending. When including the 26 patients who prematurely discontinued treatment, and excluding the patients who were either still on treatment or whose SVR status was pending, the intent to treat SVR rate for the sample was 49% (32/65). Bivariate correlates of SVR included age and HCV treatment adherence; those who achieved SVR were younger (mean age = 45.9 versus 51.7; *P* < .01) and more likely to report good HCV treatment adherence (81% versus 59%; *P* < .05) (see Table 3).

## 4. Discussion

Slightly over 40% of our sample had received HCV treatment, a much higher rate than treatment rates reported in other studies of HIV coinfected patients [4–7], in part because our rates reflect lifetime history of treatment whereas many other studies report an evaluation of treatment eligibility and initiation at a single time point. With the significant proportion of the sample having received HCV treatment, we were able to examine the frequency and correlates of treatment adherence, completion and virologic response. 

Unlike adherence to ART, which has been subject to extensive research over the past 15 years and has become known as the Achilles Heal of ART [33], this study is among the few that have examined dose-taking adherence to PEG-IFN/RBV. The study findings suggest that most HIV coinfected patients for whom treatment is recommended by the physician and who actually initiate therapy are able to adhere very well to HCV treatment, and good HCV dose-taking adherence was significantly associated with both HCV treatment completion and SVR. Also, SVR rates were higher than what has been reported in most industry clinical trials [8–10] or other community samples [11–13]. Nonetheless, a sizeable minority of patients reported missing medication doses and prematurely discontinuing treatment.

Just under 30% of the sample reported problems with missed doses of PEG-IFN/RBV, which is similar to what has been found in other studies of HCV treatment adherence [22, 23]. While our data revealed a significant relationship between HCV dose adherence and SVR, the level of dose adherence needed to achieve SVR remains unknown. Hopefully, with the pending emergence of protease inhibitor-based HCV treatments and associated vulnerability to drug resistance, research addressing this question will receive greater emphasis. 

High rates of treatment discontinuation were observed, but most stopped treatment early due to virologic non-response. Fourteen percent of treated patients stopped treatment prematurely due to treatment side effects, which is a lower rate than those reported in the literature, even though our sample included difficult-to-treat patients (e.g., current psychiatric or substance abuse problems) who are typically excluded from clinical trials [8–10]. Our findings, together with the improved ability to manage treatment side effects with aggressive use of pharmacologic agents (e.g., antidepressants, growth factors, and erythropoietin) [25, 27, 34], and higher SVR rates observed in recent studies [11], are evidence of the viability of HCV treatment for a larger, more generalized pool of coinfected patients. 

While our data generally suggest good levels of adherence, treatment completion, and even virologic response, it is important to acknowledge that most of our sample had ended their course of treatment prior to being surveyed for this study, which limits our ability to draw inferences from these data with regard to outcomes of HCV treatment. It is quite possible that patients who have successful experiences and outcomes from treatment are more likely to remain engaged in care and thus be accessible for recruitment into this study, whereas patients who discontinued treatment or were nonresponders may be more likely to drop out of care and thus not to be represented in our sample. 

Studies have been mixed with regard to the effects of depression and substance abuse on HCV treatment adherence, completion, and response [26, 27, 35]. Our data showed that having a current psychiatric disorder was associated with lower adherence. Having a psychiatric disorder was not significantly associated with treatment completion or response, but this was likely due to the small sample size and low statistical power; those who completed and responded to treatment had lower numerical rates (by 10–15%) of active psychiatric disorders. Similarly, alcohol and drug use was not associated with any of the treatment outcomes, though rates of alcohol problems were numerically less among those who adhered well to and completed treatment. While these findings are preliminary and further research is needed to more fully investigate these relationships, the data suggest that clinicians should consider incorporating brief validated measures of mental health and substance use as measures of treatment readiness. It is also noteworthy that several patients in our sample who had depression, other psychiatric disorders, and substance use problems were among those who adhered well, completed treatment, and achieved an SVR. These findings provide further support for HCV treatment recommendations that view such patients as worthy of being considered appropriate treatment candidates and advocate for treatment decisions to be made on an individual case basis [36], as opposed to excluding them from treatment as a rule. 

Aside from the limitations noted above, there are others that need to be considered. We did not randomly select patients to participate, but we did seek to enroll all coinfected patients who were consecutively attending the study sites during the 4-month recruitment period. Our measure of adherence is limited by our reliance on self-reports, rather than objective measures; self-reported adherence measures have been found to be significantly associated with HIV virologic outcomes [37], but we are not aware of such a relationship being documented with HCV virologic response. As previously alluded to, our overall measurement was hampered by needing to rely on a combination of retrospective data from clinic charts (the quality of which is dependent on the completeness of documentation and updated assessments by the clinicians) and currently assessed survey data, rather than prospectively examining adherence and its correlates from the point of treatment initiation. Furthermore, our analysis did not include measures of fibrosis and other biomarkers that could contribute to our understanding of which variables most influence treatment outcomes.

In conclusion, the data from this study provide evidence that HIV coinfected patients generally adhere well to HCV treatment and many are able to achieve sustained virologic response. Nevertheless, problems with missed doses, premature discontinuation, and nonresponse were not uncommon, and HCV dose-taking adherence was significantly associated with both treatment completion and sustained virologic response. With emerging protease inhibitor HCV treatments likely to be more effective, yet also more complex and expensive, more susceptible to resistance development, and possibly more toxic, how patients will weigh the costs and benefits of treatment, and the impact on patient adherence and treatment outcomes is unclear. Greater emphasis on research to examine adherence and its determinants is needed to optimize HCV treatment outcomes among HIV co-infected patients.

## Figures and Tables

**Table 1:** Patient characteristics and HCV treatment adherence.

Variable	Good adherence (*N* = 49)	Poor adherence (*N* = 19)	*P*-value
Demographics			
Male gender	94%	95%	0.89
African-American	27%	42%	0.21
Mean age (years)	48.5	47.1	0.59
Any college education	61%	47%	0.30
Employed	31%	42%	0.37
In a relationship	23%	26%	0.77

HIV Disease characteristics			
Mean CD4 count (cells/mm^3^)	522	560	0.50
Mean log_10_ HIV RNA (copies/ml)	2.16	2.37	0.48
HIV RNA ≤400	83%	72%	0.35

HCV Disease characteristics			
HCV genotype 1 or 4	69%	74%	0.73
Mean log_10_ HCV RNA (copies/ml)	6.04	6.22	0.46
HCV RNA <800,000	42%	44%	0.92

Psychosocial functioning			
Depressed	35%	50%	0.26
Any psychiatric disorder*	38%	67%	0.04
Any drinking problem	14%	21%	0.50
Any illicit drug use	14%	11%	0.68
Any missed appointments (past 6 months)	31%	32%	0.94
Good ART adherence^1^	60%	54%	0.69

*P < 0.05.

^1^Good adherence is defined as 100% adherence.

**Table 2:** Patient characteristics and HCV treatment completion.

Variable	Treatment complete (*N* = 43)	Treatment discontinued (*N* = 26)	*P*-value
Demographics			
Male gender	93%	96%	0.59
African American	30%	31%	0.96
Mean age (years)	47.2	50.9	0.12
Any college education	58%	58%	0.97
Employed	37%	31%	0.59
In a relationship	24%	19%	0.66

HIV Disease characteristics			
Mean CD4 count (cells/mm^3^)	507	534	0.65
Mean log_10_ HIV RNA (copies/ml)	2.29	2.17	0.63
HIV RNA ≤400	79%	77%	0.81

HCV Disease characteristics			
HCV genotype 1 or 4	65%	81%	0.17
Mean log_10_ HCV RNA (copies/ml)	6.08	6.24	0.39
HCV RNA <800,000	45%	38%	0.57

Psychosocial functioning			
Depressive disorder	36%	50%	0.26
Any psychiatric disorder	40%	58%	0.15
Any drinking problem	12%	23%	0.21
Any illicit drug use	12%	15%	0.65
Any missed clinic appointments (past 6 months)	35%	23%	0.30
Good HCV treatment adherence^∗1^	79%	55%	0.04

*P < 0.05.

^1^Good adherence is defined as 100% adherence.

**Table 3:** Patient characteristics and sustained virologic response (SVR) to HCV treatment.

Variable	SVR (*N* = 32)	No SVR (*N* = 33)	*P*-value
Demographics			
Male gender	94%	97%	0.54
African-American	25%	30%	0.63
Mean age (years)*	45.9	51.7	0.01
Any college education	53%	58%	0.72
Employed	44%	27%	0.17
In a relationship	13%	27%	0.15

HIV Disease characteristics			
Mean CD4 count (cells/mm)	486	522	0.51
Mean log_10_ HIV RNA (copies/ml)	2.43	2.09	0.20
HIV RNA ≤400	76%	81%	0.61

HCV Disease characteristics			
HCV genotype 1 or 4	63%	79%	0.15
Mean log_10_ HCV RNA (copies/ml)	6.01	6.31	0.14
HCV RNA <800,000	46%	33%	0.31

Psychosocial functioning			
Depressive disorder	41%	47%	0.67
Any psychiatric disorder	43%	56%	0.30
Any drinking problem	16%	18%	0.78
Any illicit drug use	16%	12%	0.68
Any missed clinic appointments	31%	27%	0.72
Good HCV treatment adherence^∗1^	81%	59%	0.05

*P = 0.05

^1^Good adherence is defined as 100% adherence.
